# Physicochemical Properties and Quality of Bread Enriched with Haskap Berry (*Lonicera caerulea* L.) Pomace

**DOI:** 10.3390/molecules30193884

**Published:** 2025-09-25

**Authors:** Grażyna Cacak-Pietrzak, Agata Marzec, Kacper Onisk, Stanisław Kalisz, Wioleta Dołomisiewicz, Renata Nowak, Anna Krajewska, Dariusz Dziki

**Affiliations:** 1 Department of Food Technology and Assessment, Institute of Food Sciences, Warsaw University of Life Sciences, 159C Nowoursynowska Street, 02-776 Warsaw, Poland; grazyna_cacak_pietrzak@sggw.edu.pl (G.C.-P.); stanislaw_kalisz@sggw.edu.pl (S.K.); 2 Department of Food Engineering and Process Management, Institute of Food Sciences, Warsaw University of Life Sciences, 159C Nowoursynowska Street, 02-776 Warsaw, Poland; agata_marzec@sggw.edu.pl; 3 Faculty of Food Technology, Warsaw University of Life Sciences, 159C Nowoursynowska Street, 02-776 Warsaw, Poland; kacperonisk@gmail.com; 4 Department of Pharmaceutical Botany, Medical University of Lublin, 1 Chodzki Street, 20-835 Lublin, Poland; wioleta.dolomisiewicz@umlub.edu.pl (W.D.); renata.nowak@umlub.edu.pl (R.N.); 5 Department of Food Engineering and Machines, University of Life Sciences in Lublin, 28 Głęboka Street, 20-612 Lublin, Poland; anna.krajewska@up.lublin.pl; 6 Department of Thermal Technology, University of Life Sciences in Lublin, 31 Głęboka Street, 20-612 Lublin, Poland

**Keywords:** chemical composition, antioxidant properties, phenolic profile, texture, microstructure, sensory evaluation

## Abstract

Haskap berry (*Lonicera caerulea* L.) pomace, a by-product of juice processing, is a rich source of bioactive compounds. The aim of this study was to evaluate the effect of incorporating lyophilized and ground haskap berry pomace on the physicochemical properties of wheat bread. In addition, flour water absorption and dough rheological properties were assessed. The results demonstrated that the addition of pomace increased flour water absorption and dough stability. However, these improvements did not translate into enhanced bread quality. With increasing pomace levels in the formulation, reductions in bread volume and crumb porosity, as well as an increase in crumb firmness, were observed, which consequently lowered consumer acceptability. In contrast, the addition of pomace significantly increased the dietary fiber and ash contents of the enriched bread. Moreover, the enriched bread exhibited higher antioxidant activity and phenolic compound content, along with significant alterations in the phenolic profile. Enrichment resulted in elevated concentrations of chlorogenic acid, neochlorogenic acid, cryptochlorogenic acid, protocatechuic acid, and *p*-coumaric acid. Furthermore, the contents of flavonoid aglycones, particularly quercetin and luteolin, as well as flavonoid glycosides, especially rutin and isoquercetin, were increased. Considering the quality attributes of bread enriched with *Lonicera caerulea* pomace, together with the associated increase in bioactive compounds, its proportion in wheat flour should not exceed 2%.

## 1. Introduction

The haskap berry (*Lonicera caerulea* L.) is a fruit-bearing shrub belonging to the Caprifoliaceae family, which is native to cold-climate regions such as Siberia, Kamchatka, and northern Japan [1,2]. Nowadays, the fruits of this species have gained considerable popularity in many countries due to their distinctive sensory attributes and abundance of bioactive compounds [3]. Haskap berries are particularly rich in anthocyanins, polyphenols, vitamin C, and minerals [3,4], which confer strong antioxidant properties [5]. Owing to their beneficial health effects, including anti-inflammatory, anticancer, antiviral, and cardioprotective activities [6]. Haskap berries are increasingly utilized in the food industry, especially in juice production [7], processed products, and as ingredients in functional foods [8]. Juices obtained from these berries are especially valued for their high concentrations of anthocyanins, polyphenols, and other antioxidant compounds [9].

The juice pressing process generates a substantial amount of pomace, consisting of residual skins, seeds, and pulp. This by-product remains rich in dietary fiber and retains a significant portion of the bioactive compounds naturally present in the fruit [10]. In recent years, numerous studies have investigated the potential of incorporating pomace from various fruits into food products [11,12,13,14]. However, no studies to date have examined the use of haskap pomace for food enrichment. Haskap pomace therefore represents a promising raw material for the development of functional food ingredients. Its incorporation into formulations may improve nutritional value and technological properties while also contributing to contemporary strategies aimed at reducing agro-industrial waste and promoting sustainable food production.

Bread has long been a staple of the human diet, serving as a major source of energy, carbohydrates, and minerals. Its widespread availability, variety, and sensory appeal make it an integral part of daily nutrition across many cultures. Traditionally, however, most bread is produced from refined flour, which loses a substantial proportion of the bran and germ during milling, along with essential nutrients such as dietary fiber, B vitamins, iron, magnesium, and zinc. Consequently, although refined bread provides high energy value, it is comparatively limited in nutritional quality. In recent years, there has been a growing interest in enriching bread with health-promoting ingredients, including dietary fiber [15], seeds [16], vitamins [17], and bioactive compounds [18,19]. Particular emphasis has also been placed on the incorporation of food industry by-products, such as fruit pomace [20,21,22].

Due to its structure, widespread consumption, and ability to stabilize bioactive compounds, bread serves as an effective matrix for the enrichment of food products with vitamins, minerals, and antioxidants, while maintaining appropriate sensory and technological characteristics. Nevertheless, it should be noted that the addition of functional ingredients may also adversely affect bread quality, for example, by impairing dough rheology, reducing loaf volume, modifying crumb texture, or causing undesirable changes in color and flavor [22,23]. Therefore, the selection and application of such additives should be carefully optimized to balance nutritional benefits with consumer acceptability.

Despite extensive research on bread enrichment with various plant-based ingredients, the potential use of haskap berry pomace as a functional supplement has not yet been investigated. Incorporating this by-product into bread may represent an innovative research direction, combining enhanced nutritional value with the addition of health-promoting properties to bakery products. Accordingly, the objective of the present study was to evaluate the feasibility of enriching wheat bread with haskap berry pomace and to determine the effects of its incorporation on the physicochemical properties and consumer acceptability of the final products.

## 2. Results and Discussion

### 2.1. Chemical Composition of Raw Materials and Bread

On a dry matter basis (*DM*), wheat flour (WF) contained 0.73% ash, 2.96% dietary fiber, 12.64% protein, 1.78% fat, and 81.89% digestible carbohydrates. In comparison, lyophilized pomace derived from *Lonicera caerulea* berries (LCP) exhibited higher contents of minerals, dietary fiber, and fat, while displaying lower levels of protein and digestible carbohydrates (Table 1). Specifically, the mineral content in LCP was nearly fivefold higher than in WF, dietary fiber content was more than twelvefold higher, and fat content exceeded that of WF by more than twofold. Accordingly, the incorporation of increasing proportions of LCP into the bread formulation led to progressive rises in mineral, total dietary fiber, and fat contents, accompanied by reductions in protein and digestible carbohydrate levels. Notably, the enrichment in dietary fiber was substantial, as the fiber fraction in the fruit pomace is predominantly insoluble [24]. Even the lowest inclusion level tested (1% LCP) resulted in a statistically significant increase in fiber content relative to the control bread.

Dietary fiber is well-established as a key contributor to health, associated with a lowered risk of prevalent chronic conditions such as type 2 diabetes, cardiovascular disease, and colorectal cancer. These protective effects are largely attributable to the physicochemical characteristics of fiber, including its solubility, viscosity, and fermentability, which are crucial in digestive function and the regulation of blood glucose [25,26]. Soluble fibers can form viscous gels within the gastrointestinal tract, slowing gastric emptying and nutrient absorption, thereby enhancing postprandial glycemic control [27]. Insoluble fibers facilitate intestinal transit and promote regular bowel function, while also supporting gut health by modulating the composition and activity of the microbiota. Furthermore, the microbial fermentation of fibers in the colon generates short-chain fatty acids such as acetate, propionate, and butyrate, which exert systemic effects including the regulation of glucose metabolism, anti-inflammatory actions, and maintenance of intestinal barrier integrity [28]. Collectively, these mechanisms underscore the multifaceted role of dietary fiber in glycemic management, gut health, and chronic disease prevention, highlighting its significance as a functional component of the diet [25]. The dietary fiber content of fruit pomaces may vary considerably, ranging from approximately 35% to more than 80% on a DM basis [29]. For instance, Sójka et al. [30] demonstrated that chokeberry pomace comprises approximately 70% dietary fiber. Such variation can be attributed to multiple factors, including the type of fruit and the specific fruit components retained in the pomace, the cultivar and degree of ripeness, which influence the proportion of soluble and insoluble fiber. Additionally, the processing method—including juice extraction technique, use of enzymes, and intensity of pressing—determines the amount of residual pulp and skins in the pomace [31]. Finally, post-processing treatments, such as drying method and parameters, can further impact both the total fiber content and the characteristics of its fractions [32].

### 2.2. Flour Water Absorption and Dough Physical Properties

Farinographic assessment is widely employed to evaluate the baking performance of wheat flour. This method allows for the determination of flour water absorption, a parameter of considerable practical significance that directly affects dough and bread yield. Moreover, a farinograph records the physical properties of dough during mixing, including resistance to deformation and changes in consistency. Farinographic parameters often show significant correlations with bread quality [33].

Partial replacement of WF with LCP had a significant impact on the water absorption of the blends and the rheological properties of the dough (Table 2). Water absorption increased linearly with the level of LCP, ranging from 58.5% (C) to 60.6% (LCP6). Compared to the control, a statistically significant increase in water absorption was observed at 2% LCP and higher. The substitution of WF with LCP also resulted in prolonged dough development and stability times. These effects may be attributed to the higher fiber content of LCP compared to WF (Table 1). Fiber-rich additives generally lead to increased flour water absorption and greater dough resistance to mixing, as indicated by the sum of dough development and stability times [22,34,35]. However, this is not a universal rule. Recently, Cacak-Pietrzak et al. [36] reported that partial replacement of refined wheat flour with ground cereal coffee reduced blend water absorption, had no effect on dough development time, but significantly prolonged dough stability. These discrepancies were most likely attributable to differences in the content and fractional composition of dietary fiber [37]. Haskap berry pomace consists predominantly of water-insoluble fiber fractions, such as cellulose, hemicelluloses, and lignin. In contrast, cereal coffee contains less dietary fiber than pomace, and it is mainly composed of water-soluble type II arabinogalactans and galactomannans [38]. In the present study, substituting WF with LCP increased dough softening. At the highest level of LCP applied (6%), dough softening was more than twice that of the control. Increased dough softening indicates a weakening of gluten network strength and elasticity. As demonstrated by Nawrocka et al. [35], competition for water between fiber and gluten during dough mixing leads to partial dehydration of the gluten network and structural changes, including the formation of gluten aggregates in β-sheet conformations stabilized by hydrogen bonds, which explains the observed modifications in dough rheological properties.

### 2.3. Basic Quality Parameters of Bread

The results of bread yield, volume, crumb density, and moisture content are presented in Table 3. Bread yield increased linearly with the level of LCP addition, ranging from 145.9% (C) to 151.3% (LCP6). This effect was attributed to the higher water absorption of the blends in which a portion of WF was replaced with LCP. Compared to the control, a statistically significant increase in bread yield was observed at 5% and 6% LCP inclusion levels. On the other hand, the addition of LCP negatively affected bread volume, which decreased linearly with increasing LCP content in the formulation. Compared to the control bread (C), the volume of bread with 6% LCP (LCP6) was reduced by 32.5%. Consequently, crumb density increased from 0.26 g/cm^3^ (C) to 0.47 g/cm^3^ (LCP6). Similar changes in loaf volume and crumb density were observed in our previous studies on wheat bread enriched with black chokeberry pomace [22]. These effects were attributed to the replacement of part of the flour with non-gluten protein sources and to interactions between gluten proteins and pomace components, primarily dietary fiber. Loaf volume and crumb density are important quality parameters, as they reflect the final gas retention in bread and influence consumer preferences [39]. Consumers generally prefer well-risen bread with a larger volume and soft, fine-crumbed texture. Aleixandre et al. [40] demonstrated in vivo that loaf volume and crumb structure have minimal impact on starch digestibility and postprandial metabolic response. Partial replacement of WF with LCP did not affect the crumb moisture measured 3 h after baking. However, after 96 h of storage, the moisture content of all LCP-enriched breads was significantly higher than that of the control. These results are consistent with the findings of Renzetti et al. [41], who reported that breads enriched with high-fiber ingredients exhibit slower water loss, resulting in longer crumb freshness and delayed staling.

### 2.4. Crumb Texture

Texture constitutes a key parameter influencing consumer acceptance of bread. Bread texture is influenced by both the composition of raw materials and the applied technological process [42]. The replacement of wheat flour with LCP at levels of 2% and above significantly increased the crumb hardness of fresh bread (measured three hours after baking) (Table 4). Compared with the control sample, the crumb hardness of bread containing the highest tested level of LCP (6%) more than doubled (3.65 N vs. 8.77 N, respectively). This increase in crumb hardness can be attributed to the high dietary fiber content of LCP, which resulted in reduced loaf volume and a denser, more compact crumb structure. After 96 h of storage, staling led to a significant increase in crumb hardness across all bread samples, ranging from 7.56 N (C) to 18.95 N (LCP6).

In contrast, the substitution of wheat flour with LCP did not affect crumb elasticity, springiness, or cohesiveness. Elasticity and springiness of the crumb did not change significantly during storage, whereas the cohesiveness of all bread samples decreased by nearly half. Crumb chewiness increased linearly with the level of LCP incorporated into the bread formulation. After 96 h of storage, chewiness slightly increased, although the changes were not statistically significant.

Similar adverse effects of various plant-based additives on wheat bread texture were reported in our previous studies [42,43] and have likewise been documented by other researchers [44,45]. Bread crumb texture is primarily associated with the quantity and quality of gluten, which is responsible for gas retention during fermentation. Partial replacement of wheat flour with gluten-free raw materials reduces the gluten content, leading to decreased loaf volume and a denser, harder crumb [44]. Likewise, incorporation of black chokeberry pomace at comparable levels led to a significant increase in crumb hardness and chewiness, which was evident even at a 2% addition [22]. In contrast, in gluten-free bread, the incorporation of small amounts of berry pomace, such as raspberry or chokeberry, has relatively minor effects on crumb texture [46]. This limited impact can be explained by the absence of gluten in the matrix. In wheat-based bread, gluten forms a viscoelastic protein network that interacts with fiber and water, contributing to crumb structure, elasticity, and firmness. Gluten-free breads rely mainly on starches and hydrocolloids to maintain structure, so small amounts of berry pomace do not significantly alter the matrix. Furthermore, the distribution and water-binding properties of fiber in gluten-free dough differ from those in wheat dough, further reducing the effect of pomace on texture.

### 2.5. Bread Microstructure Results

The microstructure of bread consists of pores formed during gas release in the dough fermentation process and the retention of gas throughout fermentation and baking [47]. High-quality bread should be porous, with small and uniform pores, as such a microstructure is preferred by consumers [48].

**Figure 1:**
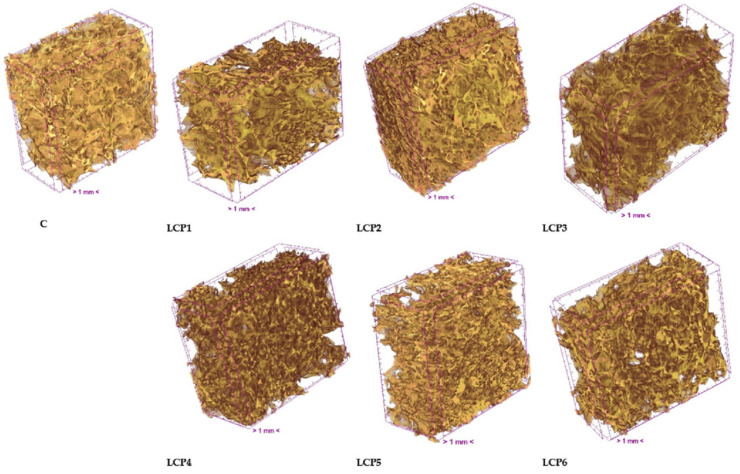
Images of the 3D microstructure of bread. C—control bread; LCP1–LCP6—bread with 1 to 6% LCP.

Figure 1 presents the 3D microstructure of bread measured using X-ray microtomography. All bread types exhibited a porous microstructure. The highest total porosity (76.78%) was observed in bread without the addition of freeze-dried haskap berry pomace (Table 5). The addition of pomace to bread increased the percent object volume while simultaneously reducing total porosity. Although both percent object volume and total porosity in bread with added freeze-dried haskap pomace changed by more than 10 percentage points compared to the control bread, these differences were not statistically significant. This lack of significance is likely due to the high standard deviation observed for measurements of bread with pomace, indicating a more heterogeneous microstructure compared to the control.

Closed porosity was low and was statistically higher (1.89%) only in bread with 2% pomace addition, compared to 0.93% in the control bread (Table 5). Open porosity is defined as the difference between total porosity and closed porosity. The microstructure of all bread samples was characterized by open porosity.

The primary parameter characterizing the average complexity of the structure is the object surface/volume ratio (OSVR) [49]. Statistically significant differences in OSVR were observed among the breads (Table 5). A significantly higher OSVR (15.60%) was recorded for bread with 2% pomace addition compared to the control. Higher levels of pomace addition had no statistically significant effect on OSVR. The degree of anisotropy (DA) is a measure of 3D structural symmetry, which in this study refers to pore arrangement. A value of 0 indicates complete isotropy, whereas a value of 1 indicates complete anisotropy [50]. The addition of pomace had no significant effect on structure thickness or DA. DA values significantly above 1 indicate an anisotropic microstructure in all bread types analyzed.

The control bread exhibited the most uniform pore size distribution, containing 80% fine pores smaller than 0.5 mm^2^, 17% pores between 0.5 and 1 mm^2^, and only 3% pores between 1 and 1.5 mm^2^. The pore surface area distribution changed significantly with the addition of LCP. In breads containing 1%, 2%, and 3% LCP, the proportion of fine pores decreased to 23%, 34%, and 52%, respectively, while the proportion of pores larger than 0.5 mm^2^ increased to 60%, 40%, and 25%. The number of large pores exceeding 1 mm^2^ rose sharply. The addition of 4%, 5%, or 6% LCP also reduced the proportion of fine pores compared to the control bread. Breads with 4%, 5%, and 6% LCP exhibited a more uniform pore size distribution than those with 1%, 2%, or 3% LCP, containing very few pores larger than 1 mm^2^, at 1%, 6%, and 1%, respectively (Figure S1).

### 2.6. Crumb Color Results

Color is an important attribute of food, enhancing its visual appeal and thereby influencing consumer preferences as well as the overall acceptance of food products. Moreover, certain natural plant pigments (e.g., anthocyanins, carotenoids) exhibit antioxidant properties; thus, color is considered to be associated with the antioxidant activity of plant-derived foods [51]. The incorporation of LCP resulted in significant alterations in bread crumb color, as evidenced by statistically significant differences in this parameter between WF and LCP formulations (Table 6). Crumb lightness (L*) decreased linearly from 64.86 (C) to 35.94 with increasing levels of LCP. The most pronounced reduction in L* was observed when wheat flour was replaced with 6% LCP (a decrease of 44.59%). This effect can be attributed to an increase in redness (a*) accompanied by a concurrent decrease in yellowness (b*). Crumb redness increased linearly from 0.62 to 11.41, whereas yellowness declined linearly from 13.61 to 4.30 with 6% substitution of wheat flour by LCP. The total color difference (ΔE) between control bread and LCP-enriched bread ranged from 9.47 to 32.24, increasing linearly with the level of LCP addition. These results demonstrate that the replacement of WF with LCP, even at a level as low as 1%, induced perceptible changes in crumb color detectable by untrained observers. LCP is a rich source of anthocyanins, particularly cyanidin-3-O-glucoside, which imparts the characteristic purple–navy hue to haskap berries [52]. Therefore, these compounds are primarily responsible for the observed changes in bread crumb coloration. Similar alterations in crumb coloration have been observed following the incorporation of freeze-dried black chokeberry pomace [22] as well as other berry-derived pomaces [46]. These effects are largely associated with the presence of anthocyanins and diverse phenolic constituents, which possess strong pigmentation and readily interact with flour components during the baking process. Beyond their direct coloring capacity, these phytochemicals may undergo thermal degradation or transformation, resulting in noticeable changes in hue and intensity. The magnitude of such alterations is influenced by factors including the botanical source of the pomace, the level of supplementation, and the specific processing conditions, underscoring the intricate interactions between plant pigments and the cereal matrix. While such visual modifications may occasionally be regarded as unfavorable in traditional bakery products, they can also serve as markers of enhanced phytochemical content and, consequently, may contribute to the improved consumer appeal of functional baked goods.

### 2.7. Antioxidant Properties of Raw Materials and Bread

The analysis of the antioxidant activity of bread enriched with LCP revealed a clear dose-dependent relationship. The control bread exhibited the highest half-maximal effective concentration values (EC50) in the scavenging ability against DPPH free radicals (DPPH), ABTS•+ cation radicals, and the ferric reducing antioxidant power (FRAP) assays, indicating the lowest antioxidant activity. The addition of LCP significantly reduced the EC50 values, with the highest activity observed in the LCP6 sample (ABTS: 30.94 ± 0.77; DPPH: 90.51 ± 2.18; FRAP: 30.89 ± 0.72 mg DM/mL) (Table 7). These results clearly indicate that LCP effectively enhances the antioxidant potential of bread, and this effect increases with higher supplementation levels. The enhancement of bread’s antioxidant activity is attributed to the high content of polyphenols and other bioactive compounds in LCP, such as phenolic acids and flavonoids (Table 8, Table 9 and Table 10), which efficiently neutralize free radicals and exhibit reducing properties. The observed decreases in EC50 values in the ABTS, DPPH, and FRAP assays confirm the synergistic effect of these compounds on the scavenging capacity toward different types of radicals and ions, which may have potential health benefits, including protection against oxidative stress. Similar results were obtained by enriching wheat bread with black chokeberry pomace, which showed enhanced antioxidant capacity as confirmed by both the ABTS and DPPH radical scavenging assays [22]. Similarly, other authors have reported increased antioxidant properties in breads enriched with pomace derived from various raw materials [53].

### 2.8. Identification and Quantification of Phenolics

Table 8 presents the concentrations of phenolic acids identified in the raw materials and enriched bread samples. In LCP, neochlorogenic acid and chlorogenic acid predominated. Chlorogenic acid and its isomer, neochlorogenic acid, are among the most important naturally occurring phenolic acids found in fruits, vegetables, and coffee. Both compounds exhibit strong antioxidant and anti-inflammatory properties, making them significant dietary constituents with potential health benefits [54]. Chlorogenic acid has been extensively studied for its effects on glucose and lipid metabolism [55], whereas neochlorogenic acid, although less widespread, demonstrates comparable biological activity, differing mainly in terms of bioavailability and chemical stability [56]. Their occurrence in plant-derived products contributes to the functional properties of foods and may support the prevention of lifestyle-related diseases [57]. Other authors observed that chokeberry and apple pomace are also significant sources of this compound [58,59]. In addition to chlorogenic and neochlorogenic acids, LCP was also found to contain cryptochlorogenic acid and protocatechuic acid. Trace amounts of *p*-coumaric acid, salicylic acid, and syringic acid were also detected. In wheat flour, among the listed compounds, chlorogenic acid, syringic acid, and protocatechuic acid were identified, with chlorogenic acid being dominant; however, its concentration was approximately 200-fold lower than in LCP.

The enrichment of bread with LCP resulted in increased concentrations of chlorogenic acid, neochlorogenic acid, cryptochlorogenic acid, protocatechuic acid, and *p*-coumaric acid. The concentration of syringic acid also increased, but only at the 1% and 2% substitution levels of wheat flour with pomace. At higher substitution levels, the control bread contained more syringic acid than the enriched samples. This phenomenon may be attributed to the binding of syringic acid to dietary fiber and proteins, which reduces the detectable amount of this compound in the final product. Moreover, both wheat flour and LCP contained only trace amounts of *p*-coumaric acid; nevertheless, in the enriched bread, this compound was detected, and its concentration was directly proportional to the proportion of LCP in the bread formulation. This can primarily be explained by the impact of the baking process and the food matrix. During thermal processing, certain phenolic compounds present in LCP become more bioaccessible, as they are released from complexes with dietary fiber and proteins, thereby increasing their detectable concentration in the final product [60]. In other words, although the raw materials initially contained only trace amounts of *p*-coumaric acid, its concentration in bread likely increased due to enhanced extraction and release during baking. *p*-Coumaric acid is a natural hydroxycinnamic acid found in many plants, including cereal grains, fruits, and vegetables. It exhibits strong antioxidant and anti-inflammatory properties and may contribute to cellular protection against oxidative stress, as well as modulate the activity of enzymes involved in lipid metabolism [61].

When analyzing the flavonoid aglycones identified in raw materials and enriched bread, it was observed that LCP were particularly rich in quercetin, containing more than two thousand times the amount present in wheat flour (Table 9). Other authors have also observed that black chokeberry pomace constitutes a valuable source of quercetin [58]. Moreover, the pomace contained substantial amounts of catechin and quercetin (23,750 ng/g dry mass (DM) and 19,187.5 ng/g DM, respectively), whereas wheat flour contained only trace levels. Quercetin is a naturally occurring flavonoid found in fruits, vegetables, and whole-grain products. Among flavonols, quercetin shows the greatest bioaccessibility in both fruits and pomace, with black chokeberry pomace demonstrating that it undergoes little degradation during digestion [58]. It exhibits strong antioxidant and anti-inflammatory properties, supports cardiovascular health as well as glucose and lipid metabolism, and may also modulate immune responses [62]. Catechin likewise displays potent antioxidant and anti-inflammatory activities, with potential cardioprotective effects [63]. The incorporation of LCP into bread led to increased levels of quercetin, catechin, and lutein. Interestingly, certain compounds, such as taxifolin, eriodictyol, and isorhamnetin, were not detectable in the pomace itself but appeared in bread at higher LCP supplementation levels (2–4%). This phenomenon is most likely attributable to the baking process, which facilitates the release of these compounds from complexes with dietary fiber and proteins [64].

When analyzing the flavonoid glycosides identified in raw materials and enriched bread, it was found that LCP were particularly rich in rutin and isoquercetin, whereas wheat flour contained only minor amounts of rutin—more than 400-fold lower than the concentration observed in LCP. High contents of these compounds have also been reported in pear pomace [65]. Rutin, also referred to as vitamin P or rutoside, has been investigated for a wide range of pharmacological properties, including antioxidant, anti-inflammatory, vasoprotective, cardioprotective, antithrombotic, and neuroprotective effects, making it a compound of considerable interest in both nutritional and therapeutic contexts [66,67]. Recent studies further indicate that isoquercetin exerts beneficial effects against neurodegenerative disorders [68] as well as cancer-related diseases [69].

In addition, the following flavonoid glycosides were identified in the pomace: isoquercetin, luteolin-7-glucoside, narcissoside, kaempferol-3-rutinoside, naringenin-7-O-glucoside, and narirutin. None of these compounds were detected in wheat flour. Substitution of wheat flour with as little as 1% LCP resulted in the appearance of rutin, narcissoside, kaempferol-3-rutinoside, and luteolin-7-glucoside in bread, with their concentrations increasing proportionally with the level of LCP supplementation. At 2% LCP substitution, all of the aforementioned flavonoid glycosides were detected, except for narirutin, which was only observed at 4% substitution. As in the pomace, rutin and isoquercetin were the most abundant compounds in the enriched bread.

A substantial body of scientific research underscores the health-promoting properties of phenolic compounds and dietary fiber. These bioactive constituents frequently co-occur in plant-derived foods, especially in pomace, thereby providing opportunities for interactions [70]. Such interactions may involve both reversible noncovalent bonds and predominantly irreversible covalent linkages, forming the basis of the so-called carrier effect of dietary fiber in the gastrointestinal tract. Through this mechanism, fiber can modulate the release, stability, and overall bioavailability of phenolics, thereby playing a key role in their physiological and health-promoting effects [71].

In the case of bread fortified with LCP, these fiber–phenolic interactions are particularly significant. LCP is a rich source of both dietary fiber and a wide spectrum of polyphenolic compounds. Within the bread matrix, phenolics can associate with both soluble and insoluble fiber fractions via hydrogen bonding, hydrophobic interactions, and covalent ester linkages. These associations can markedly influence the release and bioaccessibility of phenolics during digestion [72].

While the binding of haskap phenolics to fiber may reduce their immediate release in the upper gastrointestinal tract, potentially lowering apparent bioavailability and underestimating antioxidant activity if only small-intestinal absorption is considered, these interactions may also be advantageous. Fiber-bound phenolics are more likely to reach the colon intact, where microbial fermentation can cleave the complexes, liberating phenolics and producing smaller metabolites, such as phenolic acids, which exert both local and systemic effects [73].

The functional outcome largely depends on the physicochemical characteristics of the bread matrix. Soluble fibers in the pomace can form viscous gels that entrap different phenolics, whereas insoluble fibers (e.g., cellulose, hemicellulose, lignin) may act as carriers, physically embedding polyphenols [74]. Moreover, the molecular size and polarity of anthocyanins and phenolic acids influence whether they remain tightly bound to fiber or are gradually released during digestion [75,76].

Furthermore, incorporating LCP into bread formulations may influence the release and bioavailability of bioactive compounds, including polyphenols and dietary fiber, through both compositional and structural modifications of the bread matrix. Thermal processing during baking can facilitate the partial release of phenolic compounds bound to plant cell walls, potentially enhancing their bioaccessibility and antioxidant activity [64]. At the same time, high temperatures may cause the degradation of thermolabile compounds, thereby reducing the nutritional potential of certain bioactives [77]. Future research should use in vitro digestion and colonic fermentation models to assess how interactions between LCP and dietary fiber influence the stability, release, and biotransformation of phenolics in enriched bread, as high fiber and polyphenol content—such as in Haskap pomace—may slow starch digestion and affect bioavailability depending on thermal, matrix, and storage conditions, highlighting the need to evaluate both bioaccessibility and bioavailability in fortified baked goods.

### 2.9. Sensory Evaluation Results

**Figure 2:**
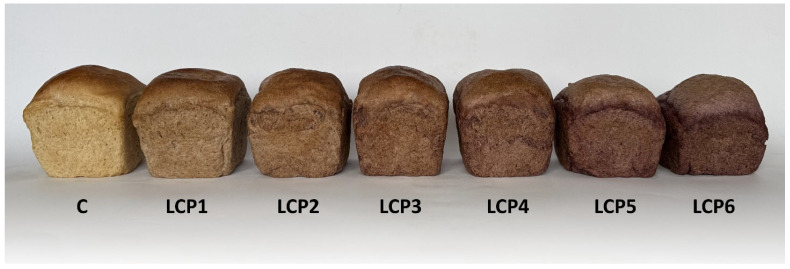
Photographs of control bread and bread enriched with LCP. C—control bread; LCP1–LCP6—bread with 1 to 6% LCP.

**Figure 3:**
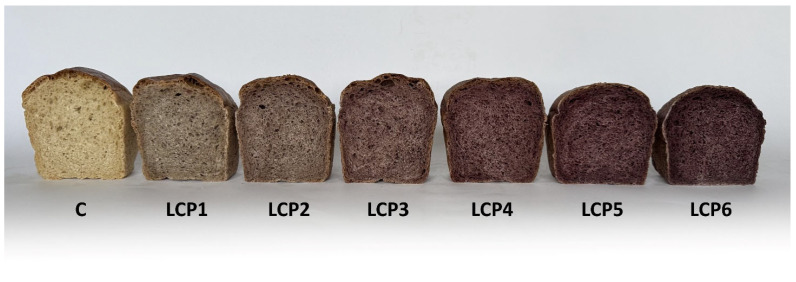
Photographs of the crumb of control bread and bread enriched with LCP. C—control bread; LCP1–LCP6—bread with 1 to 6% LCP.

Photographs of the control and enriched bread are presented in Figure 2 and Figure 3, while the results of the sensory evaluation are provided in Table 11. The highest level of acceptance in terms of the evaluated sensory attributes—appearance, smell, taste, and texture—was observed for the control bread and for the loaves enriched with 2% LCP. Increasing the LCP level in the formulation resulted in a noticeable deterioration of the bread’s external appearance. In particular, loaf volume decreased, and the crust surface became dull and deformed. These changes can be attributed to the high fiber content of the pomace, which interferes with gluten network formation and reduces gas retention capacity during fermentation and baking. As a result, the dough exhibits lower elasticity, and the final bread displays a smaller volume and a less appealing crust. In contrast, flavor and aroma were generally well accepted up to a 5% LCP level. This positive perception at lower enrichment levels may be linked to the natural fruity and slightly acidic notes imparted by LCP, which complement the typical flavor profile of wheat bread. However, at higher inclusion levels, the stronger presence of phenolic compounds and tannins likely contributed to bitterness and astringency, leading to a statistically significant reduction in consumer acceptance.

Texture acceptability was not significantly affected by the incorporation of 2% LCP. At higher levels, however, panelists reported a denser and firmer crumb structure, which negatively influenced overall texture scores. The overall acceptability results confirmed these tendencies. Incorporation of LCP up to 2% did not significantly influence consumer acceptance, whereas at 3% some panelists already rated the bread less favorably. With increasing LCP content, overall acceptance declined markedly. At 6% LCP addition, the bread was rated as “slightly disliked” by the panelists. This effect may be explained by the dilution of gluten proteins and the water-binding capacity of dietary fiber, which limit dough expansion and increase crumb compactness. Similar findings have been reported by other researchers investigating the incorporation of different types of pomace into bread [20,22,78].

These findings suggest that moderate enrichment of bread with LCP (up to 2%) can be achieved without compromising sensory quality, while higher levels of fortification—although potentially beneficial from a nutritional standpoint due to the presence of fiber and bioactive compounds—adversely affect consumer acceptance.

## 3. Materials and Methods

### 3.1. Chemicals and Raw Materials

The chemicals used in this study were previously described [79]. The following raw materials were used for dough preparation: wheat flour (WF) type 750, fresh pressed yeast, table salt, and LPC. The berry pomace used in this study was obtained from haskap berries (*Lonicera caerulea* var. kamtschatica) of the ‘Wojtek’ cultivar and represented a by-product of juice extraction performed under laboratory conditions.

### 3.2. Basic Composition of Raw Materials and Bread

The proximate composition of WF, LPC, and both control and enriched breads was analyzed following AACC standard methods [80]. The parameters determined included moisture (Method 44-15.02), ash (Method 08-01.01), total dietary fiber (Method 32-05.01), protein, and fat (Method 30.10.01). Total carbohydrate content was calculated by difference as 100 minus the sum of the other constituents.

### 3.3. Properties of Flour and Dough

The water absorption of the flour and the rheological properties of the dough were also evaluated using the Farinograph-E model 810114 (Brabender GmbH & Co. KG, Duisburg, Germany) according to AACC Method 54-21 [80]. Prior to the analysis, mixtures were prepared in which wheat flour was replaced with lyophilized pomace in amounts of 1%, 2%, 3%, 4%, 5%, and 6% relative to the flour weight. The control sample consisted of dough made from wheat flour.

### 3.4. Baking Procedure

Bread dough was prepared using the direct method according to the methodology provided by Cacak-Pietrzak et al. [22]. Wheat flour was replaced with LPC in the same amount as described in Section 3.3.

### 3.5. Basic Properties of Bread

The loaf volume was measured using a 3D scanner (NextEngine, West Los Angeles, CA, USA) with computer software (MeshLab 2016, ISTI-CNR Research Center, Rome, Italy) [22] and the crumb density of the bread was determined [81].

### 3.6. Crumb Texture

The crumb texture was assessed using a TA.XT2i texture analyzer (Stable Microsystems, Surrey, UK), following the procedure described by Armero and Collar [82]. Cylindrical samples (30 mm in diameter, 20 mm thick) were cut from bread slices and compressed using a 25 mm probe. The test consisted of two successive compressions, with a penetration depth of 40%, a 45 s interval between cycles, and a probe speed of 1 mm·s^−1^. From the resulting force–time curves, crumb hardness, elasticity, springiness, cohesiveness, and chewiness were calculated.

### 3.7. Microstructure Analysis of Crumb by X-Ray Microtomography

Bread specimens were scanned using a SkyScan 1272 micro-CT system (Bruker-microCT, Kontich, Belgium). A 180° rotational scan was performed with a 0.4° rotation step and four-frame averaging at each angular position. Each bread variant was scanned in duplicate; the cuboid samples had dimensions of 15 × 15 × 30 mm. The source–object–detector distance was adjusted to yield an isotropic voxel size of 20 µm. Scans were acquired at 50 kV and 200 µA without filtration, using a CCD camera with a 9 µm pixel resolution. A total of 470 cross-sectional images were recorded per scan.

Image reconstruction was carried out in NRecon v1.6.9.8 (Bruker-microCT, Kontich, Belgium) with Gaussian smoothing and ring-artifact correction. The resulting dataset comprised 309 grayscale tomographic slices (values 20–179), forming a three-dimensional volume. For quantitative analysis using CTAn v1.9.3.3 (Bruker-microCT, Kontich, Belgium). The 2D stack was used to reconstruct the 3D volume, and measurements were performed on volumes of interest (VOI) of 424 mm^3^. The following three-dimensional parameters were calculated:

- percent object volume (%): total volume of binarized bread structures within the VOI.
- the object surface/volume ratio (OSVR) which is the basic parameter in order to characterise the complexity of the structure and to estimate the thickness.
- total porosity: proportion of void space within the VOI.
- closed porosity: proportion of closed spaces within the VOI.
- structure thickness: average thickness of the spaces as defined by binarisation within the volume of interest images.
- degree of anisotropy (DA): measures the preferential alignment of pores.

For realistic visualization of the reconstructed microstructure, CTvol 2.3.2.0 software (Bruker-microCT, Kontich, Belgium) was employed.

### 3.8. Color Coordinates

Crumb color was assessed in the CIE-Lab* system using a CR-200 colorimeter (Konica Minolta, Osaka, Japan). Based on mean L*, a*, and b* values, the overall color difference (∆E*) between control bread and loaves enriched with LCP pomace was calculated following the method of Carvalho et al. [83].

### 3.9. Antioxidant Activity of Raw Materials and Bread

Samples weighing 1 ± 0.001 g were measured using an analytical balance and extracted with a methanol/water mixture (1:1, *v*/*v*). Extracts of the raw materials and bread samples were prepared following the procedure described by Dziki et al. [84]. The antioxidant activity of the raw materials and bread samples was evaluated. The scavenging ability against DPPH free radicals, ABTS•+ cation radicals, and the ferric reducing antioxidant power (FRAP) were determined according to the method described by Złotek et al. [79].

### 3.10. LC-ESI-MS/MS Analysis of Phenolic Acids and Flavonoid Compounds

Phenolic and flavonoid compound contents were analysed by high-performance liquid chromatography coupled with electrospray ionization mass spectrometry (LC-ESI-MS/MS), using a slightly modified method previously described by Nowacka et al. [85] and Pietrzak et al. [86]. An Agilent 1200 Series HPLC system (Agilent Technologies, Santa Clara, CA, USA) connected to a 3200 QTRAP Mass spectrometer (AB Sciex, Framingham, MA, USA) with an electrospray ionisation source (ESI) operating in negative-ion mode. Analyses were used for all analytes. Both were controlled with Analyst 1.5 software (AB Sciex, Framingham, MA, USA), which was also used for data interpretation.

Separation of phenolic and flavonoid compounds was carried out at 25 °C, on a Zorbax SB-C18 column (2.1 × 100 mm, 1.8 mm particle size; Agilent Technologies, Santa Clara, CA, USA). The mobile phase consisted of 0.1% aqueous formic acid (solvent A) and acetonitrile with 0.1% formic acid (solvent B). The injection volume was 3 µL and the flow rate was 300 µL/min. The gradient was changed as follows: 0–2 min—20% B; 3–4.5 min—25% B; 5.5–7 min—35% B; 8–12 min—65% B; 14–16 min—80% B. The total run time was 28 min.

The ESI-MS worked in negative ion mode, and the parameters were as follows: capillary temperature 550 °C, curtain gas at 30 psi, nebulizer gas at 50 psi, source voltage—4500 V.

Triplicate injections were made for each standard solution and sample. The limits of detection (LOD) and quantification (LOQ) for all analytes were determined at a signal-to-noise ratio of 3:1 and 10:1, respectively. Qualitative identifications of compounds were by comparison of MS/MS spectra and LC retention time with the corresponding standards tested under the same conditions. The calibration curves obtained in MRM mode were used for the quantification of all analytes. Detailed conditions of LC-MS analysis are given in Tables S1 and S2.

### 3.11. Sensory Evaluation

Sensory evaluation of the bread was performed 24 h after baking, following Wichchukit et al. [87]. A panel of 52 assessors (36 women and 16 men, aged 20–57 years) who regularly consumed wheat bread participated in the study. The samples were presented in a randomized order and evaluated under standardized laboratory conditions with daylight lighting and room temperature (23 °C). Panelists rated the breads on a 9-point hedonic scale. Assessed attributes included loaf and crust appearance, crumb characteristics (color, texture, porosity), taste, aroma, and overall acceptability. Water was provided between samples to cleanse the palate and minimize sensory fatigue.

### 3.12. Statistical Analysis

All measurements were carried out at least in triplicate. Statistical analysis was performed using Statistica 13.3 software (TIBCO Software, Palo Alto, CA, USA). Differences among groups were assessed by analysis of variance (ANOVA), followed by Tukey’s test to identify homogeneous subsets. Additionally, comparisons between raw materials, such as wheat flour and haskap berry pomace, were evaluated using a *t*-test. All analyses were conducted at a significance level of α = 0.05.

## 4. Conclusions

The incorporation of lyophilized haskap berry pomace into wheat bread formulations significantly affected both technological and nutritional characteristics. Although the pomace enhanced flour water absorption and dough stability, these effects did not translate into improved baking performance, as higher supplementation levels were associated with reduced loaf volume, a denser crumb structure, decreased porosity, and increased hardness, ultimately lowering consumer acceptance. In contrast, the enrichment of bread with haskap pomace provided notable nutritional benefits. The fortified loaves contained significantly higher levels of dietary fiber and minerals. Moreover, the inclusion of pomace markedly enhanced the antioxidant capacity of the bread, which corresponded with an increase in total phenolic compounds. Importantly, enrichment also resulted in a distinct modification of the phenolic profile, characterized by markedly elevated concentrations of hydroxycinnamic acids—most prominently chlorogenic, neochlorogenic, cryptochlorogenic, protocatechuic, and *p*-coumaric acid—as well as a substantial accumulation of flavonoids, both in the form of aglycones (quercetin and luteolin) and glycosides (rutin and isoquercetin). These compounds constituted the dominant fraction of the phenolic matrix, underscoring their pivotal role in shaping the overall profile. Such modifications are of particular relevance from a functional food perspective, as they may underpin potential health-promoting properties.

Considering both the sensory limitations and the nutritional benefits, the optimal incorporation level of haskap berry pomace in wheat bread formulations should be approximately 2%. At this level, the product offers enhanced health-promoting properties without substantially affecting consumer acceptance.

## Figures and Tables

**Table 1:** Basic chemical composition (% DM) of raw materials, control bread and bread enriched with LCP.

Sample	Ash	Dietary Fiber	Protein	Fat	AC
WF	0.73 ± 0.03 ^A^	2.96 ± 0.04 ^A^	12.64 ± 0.90 ^B^	1.78 ± 0.02 ^A^	81.89
LCP	3.48 ± 0.04 ^B^	36.18 ± 0.10 ^B^	10.25 ± 0.82 ^A^	4.24 ± 0.13 ^B^	45.85
C	0.75 ± 0.02 ^a^	3.02 ± 0.03 ^a^	13.20 ± 0.10 ^c^	1.84 ± 0.02 ^a^	81.19
LCP1	0.77 ± 0.02 ^ab^	3.35 ± 0.05 ^b^	13.17 ± 0.06 ^bc^	1.86 ± 0.04 ^ab^	80.85
LCP2	0.81 ± 0.05 ^abc^	3.68 ± 0.02 ^c^	13.15 ± 0.10 ^bc^	1.89 ± 0.03 ^ab^	80.47
LCP3	0.84 ± 0.03 ^bcd^	4.02 ± 0.10 ^d^	13.10 ± 0.09 ^ab^	1.93 ± 0.03 ^bc^	80.11
LCP4	0.86 ± 0.06 ^cd^	4.35 ± 0.06 ^e^	13.08 ± 0.06 ^ab^	1.94 ± 0.04 ^bc^	79.77
LCP5	0.87 ± 0.02 ^cd^	4.68 ± 0.08 ^f^	13.05 ± 0.13 ^a^	1.98 ± 0.02 ^cd^	79.42
LCP6	0.93 ± 0.05 ^d^	5.01 ± 0.04 ^g^	13.04 ± 0.12 ^a^	2.04 ± 0.03 ^d^	78.98

AC—available carbohydrates; DM—dry matter; WF—wheat flour; LCP—lyophilized *Lonicera caerulea* berry pomace; C—control bread; LCP1–LCP6—bread with 1% to 6% LCP. Data are presented as means ± standard deviations (*n* = 3). Values within a column followed by different superscript letters are significantly different (*p* < 0.05).

**Table 2:** Water absorption of flour samples and physical properties of control wheat dough and dough enriched with LCP.

Sample	Water Absorption (%)	Development Time (min)	Stability (min)	Degree of Softening (FU)
C	58.5 ± 0.0 ^a^	5.0 ± 0.5 ^c^	6.8 ± 0.5 ^a^	65 ± 6.2 ^a^
LCP1	59.0 ± 0.4 ^ab^	5.1 ± 0.6 ^a^	6.9 ± 0.6 ^ab^	95 ± 5.4 ^b^
LCP2	59.2 ± 0.1 ^b^	5.5 ± 0.1 ^b^	7.2 ± 0.3 ^ab^	111 ± 2.5 ^bc^
LCP3	59.4 ± 0.1 ^bc^	5.7 ± 0.1 ^bc^	7.3 ± 0.1 ^b^	118 ± 2.1 ^c^
LCP4	59.8 ± 0.1 ^cd^	5.9 ± 0.3 ^bc^	8.1 ± 0.3 ^c^	130 ± 4.5 ^cd^
LCP5	60.1 ± 0.2 ^de^	5.9 ± 0.2 ^bc^	8.2 ± 0.5 ^c^	144 ± 3.6 ^d^
LCP6	60.6 ± 0.1 ^e^	6.1 ± 0.4 ^c^	8.2 ± 0.7 ^c^	148 ± 6.4 ^d^

C—control sample; LCP1–LCP6—sample with 1% to 6% LCP. Data are presented as means ± standard deviations (*n* = 3). Values within a column followed by different superscript letters are significantly different (*p* < 0.05).

**Table 3:** Key quality parameters of control bread and bread enriched with LCP.

Sample	Bread Yield (%)	Bread Volume (cm^3^/100 g)	Density (g/cm^3^)	Moisture Content (%)	
				After 3 h	After 96 h
C	145.9 ± 1.1 ^a^	378 ± 1 ^d^	0.26 ± 0.00 ^a^	45.34 ± 0.26 ^a,B^	40.17 ± 0.66 ^a,A^
LCP1	146.8 ± 0.7 ^a^	327 ± 6 ^c^	0.31 ± 0.00 ^b^	45.75 ± 0.37 ^a,B^	45.23 ± 0.24 ^b,B^
LCP2	146.9 ± 0.2 ^a^	322 ± 1 ^c^	0.34 ± 0.00 ^c^	45.63 ± 0.06 ^a,B^	45.16 ± 0.20 ^b,B^
LCP3	147.6 ± 0.1 ^ab^	297 ± 0 ^b^	0.37 ± 0.00 ^d^	45.76 ± 0.33 ^a,B^	44.91 ± 0.24 ^b,B^
LCP4	149.6 ± 0.1 ^ab^	284 ± 3 ^b^	0.39 ± 0.01 ^d^	45.18 ± 0.26 ^a,B^	45.00 ± 0.53 ^b,B^
LCP5	151.1 ± 0.6 ^b^	265 ± 6 ^a^	0.44 ± 0.01 ^e^	45.63 ± 0.06 ^a,B^	45.32 ± 0.37 ^b,B^
LCP6	151.3 ± 0.2 ^b^	255 ± 2 ^a^	0.47 ± 0.00 ^f^	45.78 ± 0.07 ^a,B^	45.17 ± 0.43 ^b,B^

C—control bread; LCP1–LCP6—bread with 1% to 6% LCP. Data are presented as means ± standard deviations (*n* = 3). Values within a column followed by different superscript letters are significantly different (*p* < 0.05); lowercase letters (^a–f^) refer to the level of LCP addition, while uppercase letters (^A,B^) denote the storage time of the bread.

**Table 4:** Textural parameters of control bread and bread enriched with LCP.

Sample	Time After Baking (h)	Hardness (N)	Elasticity (-)	Springiness (-)	Cohesiveness (-)	Chewiness (N)
C		3.65 ± 0.14 ^a,A^	0.47 ± 0.02 ^a,B^	0.96 ± 0.01 ^a,A^	0.85 ± 0.03 ^a,B^	2.96 ± 0.12 ^a,A^
LCP1		4.48 ± 0.28 ^ab,A^	0.43 ± 0.01 ^a,B^	0.93 ± 0.01 ^a,A^	0.83 ± 0.03 ^a,B^	3.46 ± 0.31 ^ab,A^
LCP2		5.45 ± 0.27 ^bc,A^	0.43 ± 0.01 ^a,B^	0.95 ± 0.02 ^a,A^	0.81 ± 0.04 ^a,B^	4.19 ± 0.42 ^bc,A^
LCP3	3	6.28 ± 0.12 ^c,A^	0.44 ± 0.03 ^a,B^	0.95 ± 0.01 ^a,A^	0.81 ± 0.04 ^a,B^	4.83 ± 0.28 ^c,A^
LCP4		8.30 ± 0.20 ^d,A^	0.42 ± 0.02 ^a,B^	0.94 ± 0.03 ^a,A^	0.78 ± 0.04 ^a,B^	6.13 ± 0.06 ^d,A^
LCP5		8.48 ± 0.32 ^d,A^	0.47 ± 0.02 ^a,B^	0.95 ± 0.06 ^a,A^	0.76 ± 0.05 ^a,B^	6.33 ± 0.45 ^d,A^
LCP6		8.77 ± 0.56 ^d,A^	0.42 ± 0.02 ^a,B^	0.94 ± 0.01 ^a,A^	0.76 ± 0.03 ^a,B^	6.23 ± 0.22 ^d,A^
C		7.56 ± 1.59 ^a,B^	0.17 ± 0.06 ^a,A^	0.90 ± 0.06 ^a,A^	0.46 ± 0.13 ^a,A^	3.19 ± 0.47 ^a,A^
LCP1		9.13 ± 0.35 ^ab,B^	0.15 ± 0.04 ^a,A^	0.91 ± 0.05 ^a,A^	0.44 ± 0.07 ^a,A^	3.70 ± 0.81 ^ab,A^
LCP2		9.49 ± 1.73 ^ab,B^	0.14 ± 0.04 ^a,A^	0.93 ± 0.04 ^a,A^	0.40 ± 0.11 ^a,A^	3.58 ± 0.34 ^ab,A^
LCP3	96	10.08 ± 1.20 ^ab,B^	0.16 ± 0.04 ^a,A^	0.85 ± 0.06 ^a,A^	0.43 ± 0.07 ^a,A^	3.63 ± 0.67 ^ab,A^
LCP4		12.88 ± 1.12 ^bc,B^	0.14 ± 0.02 ^a,A^	0.87 ± 0.03 ^a,A^	0.40 ± 0.04 ^a,A^	4.47 ± 0.06 ^b,A^
LCP5		16.15 ± 1.63 ^cd,B^	0.15 ± 0.02 ^a,A^	0.90 ± 0.06 ^a,A^	0.42 ± 0.05 ^a,A^	6.06 ± 0.45 ^bc,A^
LCP6		18.95 ± 1.71 ^d,B^	0.16 ± 0.00 ^a,A^	0.90 ± 0.06 ^a,A^	0.43 ± 0.01 ^a,A^	7.26 ± 0.49 ^c,A^

C—control bread; LCP1–LCP6—bread with 1% to 6% LCP. Data are presented as means ± standard deviations (*n* = 6). Values within a column followed by different superscript letters are significantly different (*p* < 0.05); lowercase letters (^a–d^) refer to the level of LCP addition, while uppercase letters (^A,B^) denote the storage time of the bread.

**Table 5:** Three-dimensional microstructural parameters of control bread and bread enriched with LCP.

Sample	Percent Object Volume (%)	Total Porosity (%)	Closed Porosity (%)	Object Surface/Volume Ratio (OSVR) (1/mm)	Structure Thickness (mm)	Degree of Anisotropy (DA) (-)
C	23.22 ± 0.52 ^a^	76.78 ± 0.52 ^a^	0.93 ± 0.30 ^a^	12.32 ± 1.96 ^ab^	0.307 ± 0.041 ^a^	1.93 ± 0.31 ^a^
LCP1	34.47 ± 5.93 ^a^	65.53 ± 5.93 ^a^	1.38 ± 0.13 ^ab^	10.58 ± 0.44 ^a^	0.346 ± 0.015 ^a^	1.36 ± 0.14 ^a^
LCP2	33.80 ± 7.96 ^a^	66.20 ± 7.96 ^a^	1.89 ± 0.24 ^b^	15.60 ± 0.43 ^c^	0.271 ± 0.029 ^a^	1.72 ± 0.04 ^a^
LCP3	32.41 ± 3.43 ^a^	67.59 ± 3.43 ^a^	0.83 ± 0.03 ^a^	14.44 ± 0.52 ^bc^	0.295 ± 0.060 ^a^	1.65 ± 0.18 ^a^
LCP4	32.83 ± 2.61 ^a^	67.17 ± 2.61 ^a^	0.86 ± 0.11 ^a^	12.20 ± 0.22 ^ab^	0.286 ± 0.011 ^a^	1.38 ± 0.08 ^a^
LCP5	28.78 ± 1.34 ^a^	71.22 ± 1.34 ^a^	0.86 ± 0.17 ^a^	12.44 ± 0.13 ^abc^	0.255 ± 0.033 ^a^	1.86 ± 0.71 ^a^
LCP6	31.77 ± 2.43 ^a^	68.23 ± 2.43 ^a^	0.95 ± 0.05 ^a^	13.56 ± 0.35 ^abc^	0.300 ± 0.060 ^a^	1.65 ± 0.40 ^a^

C—control bread; LCP1–LCP6—bread with 1% to 6% LCP. Data are presented as means ± standard deviations (*n* = 3). Values within a column followed by different superscript letters are significantly different (*p* < 0.05).

**Table 6:** Color coordinates of raw materials and the crumb of control bread and bread enriched with LCP.

Sample	L* (-)	a* (-)	b* (-)	Total Color Difference (-)
WF	92.39 ± 0.4 ^B^	−0.13 ± 0.3 ^A^	9.88 ± 1.5 ^B^	-
LCP	24.42 ± 0.5 ^A^	14.62 ± 0.1 ^B^	1.40 ± 5.7 ^A^	-
C	64.86 ± 1.43 ^g^	0.62 ± 0.33 ^a^	13.61 ± 1.00 ^f^	-
LCP1	55.81 ± 1.14 ^f^	2.03 ± 0.36 ^b^	11.20 ± 0.52 ^e^	9.47
LCP2	52.38 ± 1.13 ^e^	3.12 ± 0.20 ^c^	10.20 ± 0.51 ^d^	13.17
LCP3	46.83 ± 0.83 ^d^	4.19 ± 0.36 ^d^	8.56 ± 0.72 ^c^	19.22
LCP4	44.08 ± 0.95 ^c^	7.80 ± 0.35 ^e^	7.72 ± 0.51 ^c^	22.76
LCP5	39.85 ± 0.59 ^b^	9.25 ± 0.54 ^f^	5.50 ± 0.60 ^b^	27.67
LCP6	35.94 ± 0.55 ^a^	11.41 ± 0.33 ^g^	4.30 ± 0.40 ^a^	32.24

WF—wheat flour; LCP—lyophilized *Lonicera caerulea* berry pomace; C—control bread; LCP1–LCP6—bread with 1% to 6% LCP. Data are presented as means ± standard deviations (*n* = 10). Values within a column followed by different superscript letters are significantly different (*p* < 0.05).

**Table 7:** Antioxidant activity (EC50 index) in raw materials, control bread and bread enriched with LCP.

Sample	ABTS_EC50_ (mg DM/mL)	DPPH_EC50_ (mg DM/mL)	FRAP_EC50_ (mg DM/mL)
WF	598.21 ± 7.94 ^B^	623 ± 11.75 ^B^	732 ± 6.38 ^B^
LCP	6.14 ± 0.26 ^A^	11.53 ± 0.45 ^A^	5.27 ± 0.22 ^A^
C	114.35 ± 2.26 ^g^	885.45 ± 10.47 ^f^	191.00 ± 5.06 ^f^
LCP1	77.93 ± 1.47 ^f^	177.18 ± 1.70 ^e^	115.37 ± 3.03 ^e^
LCP2	56.01 ± 0.77 ^e^	125.86 ± 1.18 ^d^	71.57 ± 0.83 ^d^
LCP3	46.15 ± 1.00 ^d^	110.41 ± 2.27 ^c^	54.04 ± 1.01 ^c^
LCP4	41.99 ± 0.76 ^c^	105.68 ± 0.96 ^bc^	43.56 ± 1.38 ^b^
LCP5	36.61 ± 0.45 ^b^	96.58 ± 1.42 ^ab^	36.96 ± 1.03 ^ab^
LCP6	30.94 ± 0.77 ^a^	90.51 ± 2.18 ^a^	30.89 ± 0.72 ^a^

WF—wheat flour; LCP—lyophilized *Lonicera caerulea* berry pomace; C—control bread; LCP1–LCP6—bread with 1% to 6% LCP. Data are presented as means ± standard deviations (*n* = 3). Values within a column followed by different superscript letters are significantly different (*p* < 0.05).

**Table 8:** Phenolic acids (ng/g DM) identified in raw materials, control bread and bread enriched with LCP.

Sample	Gallic Acid	Protocatechuic Acid	*p*-Coumaric Acid	Salicylic Acid	Syringic Acid	Chlorogenic Acid	NEO	CRY
WF	trace	177.3 ± 1.0 ^A^	trace	trace	184.67	1133.3 ± 14.4 ^A^	trace	nd
LCP	nd	700.6 ± 7.9 ^B^	trace	trace	trace	224,375 ± 883.8 ^B^	47,375 ± 187.0	2375 ± 53.6
C	trace	281.4 ± 2.4 ^a^	trace	trace	381.8 ± 3.3 ^e^	535.2 ± 5.6 ^a^	trace	trace
LCP1	trace	3532.7 ± 80.1 ^b^	23.40 ± 0.64 ^a^	trace	714.2 ± 13.1 ^g^	4809.9 ± 54.7 ^b^	163.3 ± 1.8 ^a^	107.1 ± 4.2 ^a^
LCP2	trace	9642.2 ± 39.4 ^c^	52.12 ± 2.02 ^b^	trace	466.27 ± 3.9 ^f^	14,721.5 ± 12.1 ^c^	568.3 ± 3.6 ^b^	285.2 ± 8.7 ^b^
LCP3	trace	11,453.3 ± 13.38 ^d^	71.88 ± 0.23 ^c^	trace	349.4 ± 9.0 ^d^	19,453.8 ± 61.4 ^d^	724.5 ± 21.0 ^c^	333.3 ± 10.4 ^c^
LCP4	trace	17,005.6 ± 41.18 ^e^	89.01 ± 0.26 ^d^	trace	322.6 ± 10.5 ^c^	28,190.6 ± 42.0 ^e^	1458.0 ± 4.4 ^d^	427.7 ± 5.9 ^e^
LCP5	trace	19,556.5 ± 31.8 ^f^	103.47 ± 1.94 ^e^	trace	170.03 ± 2.7 ^b^	31,351.6 ± 53.3 ^f^	1786.2 ± 12.2 ^e^	458.6 ± 3.2 ^e^
LCP6	trace	21,084.5 ± 72.8 ^g^	112.81 ± 1.02 ^f^	trace	135.1 ± 1.0 ^a^	35,322.4 ± 38.6 ^g^	1869.7 ± 48.6 ^f^	453.8 ± 27.5 ^e^

WF—wheat flour; LCP—lyophilized *Lonicera caerulea* berry pomace; C—control bread; LCP1–LCP6—bread with 1% to 6% LCP; NEO—neochlorogenic acid; CRY—Cryptochlorogenic acid; nd—not detected. Data are presented as means ± standard deviations (*n* = 3). Values within a column followed by different superscript letters are significantly different (*p* < 0.05).

**Table 9:** Flavonoid aglycones (ng/g DM) identified in raw materials, control bread and bread enriched with LCP.

Sample	Apigenin	Luteolin	Eriodictyol	Quercetin	Catechin	Isorhamnetin	Taxifolin
WF	nd	trace	nd	8.67 ± 0.19 ^A^	trace	nd	nd
LCP	trace	345.75 ± 5.4	trace	19,187.50 ± 97.62 ^B^	23,750 ± 278.2	trace	521.88 ± 9.72
C	trace	18.64 ± 0.87 ^a^	nd	nd	nd	nd	nd
LCP1	trace	35.47 ± 0.70 ^b^	trace	293.1 ± 2.0 ^a^	494.8 ± 2.7 ^a^	nd	trace
LCP2	trace	57.75 ± 1.34 ^c^	trace	660.1 ± 1.9 ^b^	1657.1 ± 14.5 ^b^	trace	176.36 ± 1.04 ^a^
LCP3	trace	86.30 ± 1.09 ^d^	9.43 ± 0.37 ^a^	1355.7 ± 6.6 ^c^	2247.6 ± 2.1 ^c^	trace	223.18 ± 5.33 ^b^
LCP4	trace	126.55 ± 3.17 ^e^	78.38 ± 1.59 ^b^	3245.0 ± 3.9 ^d^	3456.6 ± 39.6 ^d^	144.83 ± 9.72 ^a^	344.20 ± 2.22 ^d^
LCP5	trace	241.01 ± 1.65 ^f^	91.41 ± 1.50 ^c^	8643.6 ± 47.8 ^e^	3317.4 ± 15.7 ^e^	217.83 ± 13.08 ^b^	383.47 ± 2.38 ^e^
LCP6	trace	323.57 ± 7.08 ^g^	112.42 ± 2.53 ^d^	5429.1 ± 38.4 ^f^	3900.3 ± 51.9 ^f^	240.07 ± 7.26 ^c^	329.65 ± 0.57 ^c^

WF—wheat flour; LCP—lyophilized *Lonicera caerulea* berry pomace; C—control bread; LCP1–LCP6—bread with 1% to 6% LCP; nd—not detected. Data are presented as means ± standard deviations (*n* = 3). Values within a column followed by different superscript letters are significantly different (*p* < 0.05).

**Table 10:** Flavonoid glycosides (ng/g DM) identified in raw materials, control bread and bread enriched with LCP.

Sample	Naringenin 7-O-Glucoside	Isoquercetin	Luteolin-7-Glucoside	Narirutin	Kaempferol-3-Rutinoside	Rutin	Narcissoside
WF	nd	nd	nd	nd	nd	608.3 ± 30.6 ^A^	nd
LCP	6443.8 ± 203.3	123,625 ± 707	38,125 ± 325	5562.5 ± 17.7	20,062.5 ± 88.39	251,875 ± 2651 ^B^	44,187.5 ± 265.2
C	nd	nd	nd	nd	nd	nd	trace
LCP1	nd	trace	57.62 ± 0.52 ^a^	trace	174.9 ± 3.6 ^a^	3523.0 ± 179.2 ^a^	643.9 ± 10.0 ^a^
LCP2	131.91 ± 1.78 ^a^	113.0 ± 2.5 ^a^	105.96 ± 3.99 ^b^	trace	696.2 ± 6.3 ^b^	12,820.0 ± 139.2 ^b^	1756.7 ± 24.1 ^b^
LCP3	222.62 ± 6.93 ^b^	807.5 ± 11.7 ^b^	189.34 ± 2.73 ^c^	trace	813.3 ± 7.8 ^c^	17,267.9 ± 348.3 ^c^	2183.2 ± 7.9 ^c^
LCP4	351.72 ± 8.92 ^c^	3127.8 ± 20.5 ^c^	489.20 ± 6.35 ^d^	144.33 ± 1.95 ^a^	1410.3 ± 7.1 ^d^	26,237.8 ± 55.2 ^d^	3548.9 ± 15.9 ^d^
LCP5	455.49 ± 2.90 ^e^	4584.8 ± 165.9 ^d^	603.14 ± 4.37 ^e^	217.75 ± 2.18 ^b^	1862.0 ± 13.5 ^e^	31,229.0 ± 60.7 ^e^	3739.7 ± 130.5 ^e^
LCP6	386.42 ± 3.13 ^d^	5584.8 ± 82.9 ^e^	1071.90 ± 17.68 ^f^	217.36 ± 10.92 ^b^	1791.2 ± 5.6 ^e^	31,325.4 ± 63.1 ^e^	4003.6 ± 46.0 ^e^

WF—wheat flour; LCP—lyophilized *Lonicera caerulea* berry pomace; C—control bread; LCP1–LCP6—bread with 1% to 6% LCP; nd—not detected. Data are presented as means ± standard deviations (*n* = 3). Values within a column followed by different superscript letters are significantly different (*p* < 0.05).

**Table 11:** Sensory evaluation results of control bread and bread enriched with LCP.

Sample	Appearance (pt)	Smell (pt)	Taste (pt)	Texture (pt)	Overall Acceptability (pt)
C	7.37 ± 0.71 ^d^	7.83 ± 0.88 ^c^	7.54 ± 0.50 ^cd^	7.76 ± 0.79 ^e^	7.57 ± 0.49 ^e^
LCP1	7.22 ± 0.70 ^cd^	7.63 ± 0.77 ^bc^	7.70 ± 0.63 ^bd^	7.67 ± 0.60 ^e^	7.55 ± 0.35 ^e^
LCP2	7.11 ± 0.67 ^cd^	7.57 ± 0.68 ^bc^	7.61 ± 0.58 ^cd^	7.41 ± 0.62 ^de^	7.34 ± 0.35 ^e^
LCP3	6.83 ± 0.64 ^c^	7.37 ± 0.69 ^b^	7.63 ± 0.74 ^cd^	7.15 ± 0.76 ^d^	6.83 ± 0.52 ^d^
LCP4	4.80 ± 0.83 ^b^	7.32 ± 0.74 ^b^	6.98 ± 0.61 ^b^	6.63 ± 0.95 ^c^	6.12 ± 0.62 ^c^
LCP5	4.37 ± 0.93 ^b^	7.28 ± 0.58 ^b^	7.00 ± 0.70 ^b^	6.02 ± 0.98 ^b^	5.57 ± 0.61 ^b^
LCP6	3.07 ± 0.98 ^a^	6.46 ± 0.78 ^a^	6.24 ± 0.71 ^a^	5.41 ± 0.96 ^a^	4.40 ± 0.85 ^a^

C—control bread; LCP1–LCP6—bread with 1% to 6% LCP. Data are presented as means ± standard deviations (*n* = 52). Values within a column followed by different superscript letters are significantly different (*p* < 0.05).

## Data Availability

The original contributions presented in the study are included in the article; further inquiries can be directed to the corresponding authors.

## Supplementary Materials

The following supporting information can be downloaded at: https://www.mdpi.com/article/10.3390/molecules30193884/s1, Table S1. LC-ESI-MS/MS analytical results of phenolic acids investigated in samples. Compounds confirmed by; comparison with authentic standards. Table S2. Limit of detection (LOD), limit of quantification (LOQ) and calibration curve parameters for phenolic acids; Figure S1. The distribution of pore size in the microstructure of control bread and bread enriched with LCP.
