# Supplementary material for: Physicochemical Properties and Quality of Bread Enriched with Haskap Berry (Lonicera caerulea L.) Pomace
Source: Molecules. 2025 Sep 25;30(19):3884. doi: 10.3390/molecules30193884 (PMC12526177; doi:10.3390/molecules30193884)
Supplement: Supplementary file 1 [file molecules-30-03884-s001.zip › molecules-3875541-supplementary.pdf]

Table S1. LC-ESI-MS/MS analytical results of phenolic acids investigated in samples. Compounds confirmed by comparison with authentic standards.

Compound	Retention time [min]	$[M-H]^-$ [m/z]	Fragment ions [m/z]	Colision energy [eV]
**Phenolic acids**				
Gallic acid	5.16	168.7	78.9<br>124.9	-36<br>-14
3-O-caffeoylquinic acid (neochlorogenic acid)	6.9	353	191<br>178.9	-3<br>-3
Protocatechuic acid	8.42	152.9	80.9<br>107.8	-26<br>-38
5-caffeoylquinic acid (chlorogenic acid)	9.30<br>10.42	352.9	190.8<br>84.9	-24<br>-60
4-caffeoylquinic acid (cryptochlorogenic acid)	9.4	353	173<br>135	-3<br>-3
4-Hydroxybenzoic acid	10.84	136.8	92.9	-18
Syringic acid	11.42	196.9	122.8<br>181.9	-24<br>-12
4-Hydroxycinnamic acid (*p*-coumaric acid)	14.10	162.7	119<br>93	-14<br>-44
Salicylic acid	17.91	136.8	93<br>75	-16<br>-48
**Flavonoid aglycones**				
Catechin	9.64	288.8	244.9<br>109	-16<br>-32
Taxifolin	15.15	302.7	124.9<br>284.8	-26<br>-14
Luteolin	17.82	284.7	132.9<br>150.9	-38<br>-26
Eriodictyol	17.89	286.7	134.9<br>150.9	-32<br>-18
Quercetin	17.94	300.7	150.9<br>178.8	-26<br>-20
Apigenin	18.64	268.8	117<br>106.8	-44<br>-34
Kaempferol	18.85	284.7	116.8<br>93	-46<br>-52
Isorhamnetin	18.99	314.7	299.7<br>150.9	-20<br>-30
**Flavonoid glycosides**				
Eriodictyol-7-*O*-rutinoside (Eriocitrin)	11.93	594.8	286.9<br>150.9	-34<br>-46
Quercetin -3-*O*-rutinoside (Rutin)	11.99	608.7	299.6<br>270.9	-46<br>-60
Luteolin-7-*O*-glucoside (Luteoloside)	12.87	446.8	284.8<br>132.9	-30<br>-78
Quercetin -3-*O*-glucoside (Isoquercetin)	13.00	462.7	299.7<br>270.7	-30<br>-44
Eriodictyol-7-*O*-glucopyranoside	13.06	448.8	286.9<br>134.9	-24<br>-48

Kaempferol – 3-*O*-rutinoside (Nicotiflorin)	13.31	592.7	284.8<br>226.7	-38<br>-68
Isorhamnetin-3-*O*-rutinoside (Narcissoside)	13.52	622.8	314.9<br>298.8	-40<br>-52
Naringenin-7-*O*-rutinoside (Narirutin)	13.80	578.9	270.8<br>118.9	-34<br>-76
Kaempferol – 3-*O*-glucoside (Astragalin)	14.66	446.7	226.8<br>254.8	-54<br>-40
Isorhamnetin-3-*O*-glucoside	14.76	476.8	313.9<br>270.9	-30<br>-44
Naringenin 7-*O*-glucoside	15.12	432.7	270.8<br>118.9	-22<br>-64

Table S2. Limit of detection (LOD), limit of quantification (LOQ) and calibration curve parameters for phenolic acids.

Compound	LOD [ng/mL]	LOQ [ng/mL]	R^2	Linearity range [ng/mL]
**Phenolic acids**				
Gallic acid	1000	1850	0.9986	1850-18500
3-O-caffeoylquinic acid (neochlorogenic acid)	20	40	0.9996	40-10000
Protocatechuic acid	200	400	0.9988	1890-18900
5-caffeoylquinic acid (chlorogenic acid)	72	180	0.9991	180-18000
4-caffeoylquinic acid (cryptochlorogenic acid)	20	40	0.9979	40-4000
4-Hydroxybenzoic acid	200	250	0.9994	700-19250
Syringic acid	500	732	0.9993	732-18300
4-Hydroxycinnamic acid (*p*-coumaric acid)	83	200	0.9990	400-13800
Salicylic acid	500	700	0.9974	1800-18000
**Flavonoid aglycones**				
Catechin	10	20	0.9978	30-1500
Taxifolin	10	20	0.9981	40-10000
Luteolin	6	16	0.9974	33-1650
Eriodictyol	33	66	0.9984	66-6600
Quercetin	66	132	0.9977	132-6600
Apigenin	15	22	0.9979	89-4470
Kaempferol	33	66	0.9977	165-3300
Isorhamnetin	66	132	0.9990	132-6600
**Flavonoid glycosides**				
Eriodictyol-7-*O*-rutinoside (Eriocitrin)	250	500	0.9989	500-50000
Quercetin -3-*O*-rutinoside (Rutin)	90	180	0.9981	450-45000
Luteolin-7-*O*-glucoside (Luteoloside)	100	200	0.9983	3300-50000

Quercetin -3-*O*-glucoside (Isoquercetin)	100	250	0.9994	1670-50000
Eriodictyol-7-*O*-glucopyranoside	100	200	0.9984	2000-50000
Kaempferol – 3-*O*-rutinoside (Nicotiflorin)	167	250	0.9983	500-25000
Isorhamnetin-3-*O*-rutinoside (Narcissoside)	50	100	0.9985	1000-50000
Naringenin-7-*O*-rutinoside (Narirutin)	100	250	0.9988	1670-25000
Kaempferol – 3-*O*-glucoside (Astragalin)	50	100	0.9986	1000-25000
Isorhamnetin-3-*O*-glucoside	100	140	0.9983	2330-35000
Naringenin 7-*O*-glucoside	100	167	0.9987	250-25000

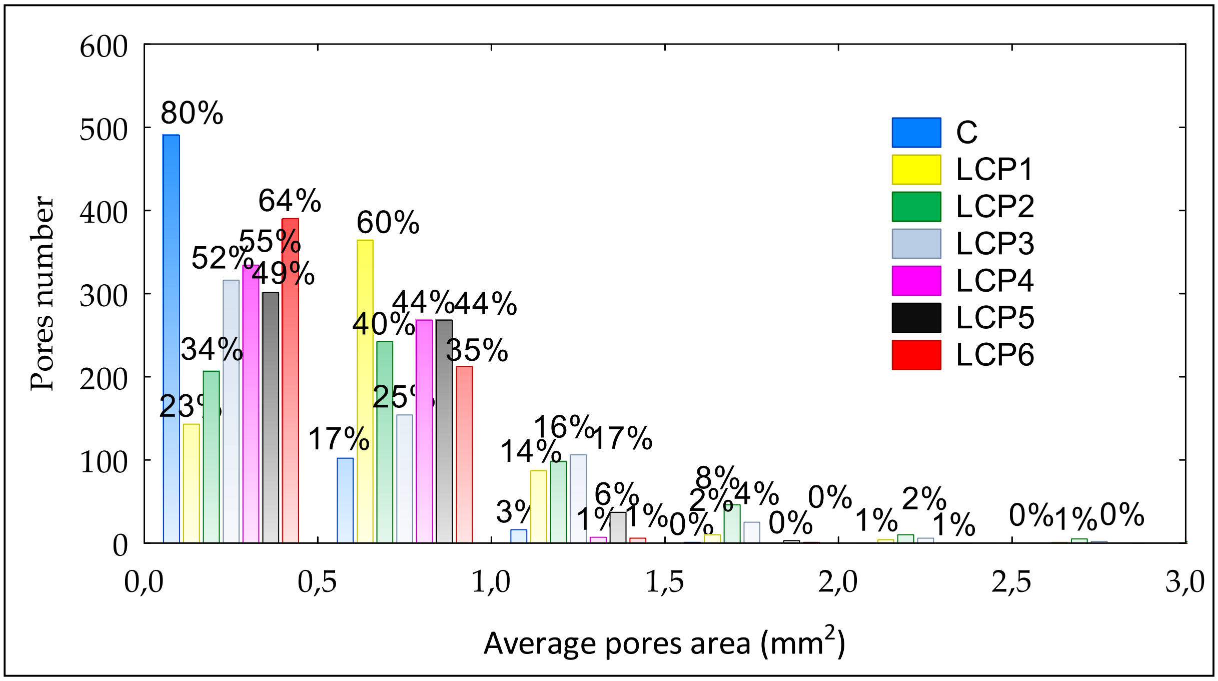

Figure S1. The distribution of pore size in the microstructure of control bread and bread enriched with LCP.
